# Pathogens in fleas collected from cats and dogs: distribution and prevalence in the UK

**DOI:** 10.1186/s13071-019-3326-x

**Published:** 2019-02-06

**Authors:** Swaid Abdullah, Chris Helps, Séverine Tasker, Hannah Newbury, Richard Wall

**Affiliations:** 1 Veterinary Parasitology and Ecology Group, School of Biological Sciences, University of Bristol, Bristol, UK; 2 Present address: School of Veterinary Science, Faculty of Science, The University of Queensland, Gatton, Australia; 3 Molecular Diagnostic Unit, Langford Vets and Bristol Veterinary School, University of Bristol, Bristol, UK; 4 Present address: The Linnaeus Group, Shirley, UK; 5 MSD Animal Health, Walton Manor, Walton, Milton Keynes, UK

**Keywords:** Companion animal, Disease, Flea-borne, Pathogen, Siphonaptera, Vector, *Bartonella*

## Abstract

**Background:**

Fleas (Siphonaptera) are the most clinically important ectoparasites of dogs and cats worldwide. Rising levels of pet ownership, climate change and globalisation are increasing the importance of a detailed understanding of the endemicity and prevalence of flea-borne pathogens. This requires continued surveillance to detect change. This study reports a large-scale survey of pathogens in fleas collected from client-owned cats and dogs in the UK.

**Methods:**

Recruited veterinary practices were asked to follow a standardised flea inspection protocol on a randomised selection of cats and dogs brought into the practice in April and June 2018. A total of 326 practices participated and 812 cats and 662 dogs were examined. Fleas were collected, identified to species and pooled flea samples from each host were analysed for the presence of pathogens using PCR and sequence analysis.

**Results:**

Overall, 28.1% of cats and 14.4% of dogs were flea infested. More than 90% of the fleas on both cats and dogs were cat fleas, *Ctenocephalides felis felis*. Fleas of the same species from each infested host were pooled. DNA was amplified from 470 of the pooled flea samples using conventional PCR, 66 of which (14% ± 95% CI 3.14%) were positive for at least one pathogen. Fifty-three (11.3% ± 95% CI 2.85%) of the pooled flea DNA samples were positive for *Bartonella* spp., 35 were from cats and 4 from dogs, the remainder had no host record. Seventeen of the *Bartonella* spp. samples were found to be *Bartonella henselae*, 27 were *Bartonella clarridgeiae* (of two different strains), 4 samples were *Bartonella alsatica* and one was *Bartonella grahamii*; 4 samples could not be identified. Fourteen (3% ± 95% CI 1.53%) of the flea DNA samples were found to be positive for *Dipylidium caninum*, 10 of the *D. caninum-*infected samples were collected from cats and one from a dog, the other 3 positive flea samples had no host species record. Only 3 flea samples were positive for *Mycoplasma haemofelis* or *Mycoplasma haemocanis*; 2 were collected from cats and one had no host species record. Three fleas were positive for both *D. caninum* and *Bartonella* spp. One flea was positive for both *Bartonella* spp. and *M. haemofelis* or *M. haemocanis*.

**Conclusions:**

This study highlights the need for ongoing flea control, particularly given the relatively high prevalence of *Bartonella* spp., which is of concern for both animal welfare and human health. The study demonstrates the ongoing need to educate pet owners about the effects of both flea infestation and also the pathogen risks these fleas present.

## Background

The promiscuous blood-feeding behaviour of both sexes of fleas (Siphonaptera), their mobility, their persistence in the environment and their ability to act as vectors of a range of pathogens all contribute to making them the most clinically important ectoparasites of dogs and cats worldwide [1]. Of particular importance for companion animals are the cat flea, *Ctenocephalides felis felis*, and the dog flea, *C. canis*, which are widely distributed globally. These species are generally considered to be host-preferential rather than host-specific and will try to feed on any available animal; *C. f. felis* has been found on over fifty different host species, which contributes to its persistence in the environment [2]. The prevalence of flea infestation in companion animals is commonly reported as varying between 10–40% [3–6], although in some instances peak infestation rates of more than 70% have been reported [6]. Infestation rates are highly variable from year to year and also depend on location, whether animals are rural or urban, lifestyle - for example outdoor access, whether they live in multi-pet households and the frequency of effective insecticidal treatments. Seasonal variations in infestation prevalence are also commonly seen, with a lower prevalence in winter and an increase from spring to autumn [7]. A knowledge of the prevalence of *C. f. felis* and *C. canis* on domestic animals and other wild hosts has important implications for flea control, since it affects the extent to which they may act as reservoirs of fleas, from which treated animals may be continuously reinfested.

Besides the direct effects resulting from blood-feeding, *Ctenocephalides* species are important as competent vectors for a wide range of pathogens, many of which are zoonotic [8–10]. In particular, these fleas may be vectors of rickettsiae, such as *Yersinia pestis*, *Rickettsia typhi*, *Rickettsia felis*, *Rickettsia conorii* and *Bartonella henselae* [11] and are the intermediate hosts for cysticercoid larvae of *Dypidilium caninum* tapeworms [12, 13]. Amongst the factors that contribute to the highly effective role of fleas as vectors includes the transovarial and transtadial transmission of some pathogens [14, 15]. *Dipylidium caninum* infection is very widely prevalent and, being dependent on the continuous presence of vectors for its local endemicity, infestations are seen in both neglected animals and owned domestic dogs and cats [16].

Several *Bartonella* spp. transmitted by fleas can induce clinical disease in both dogs and humans and these pathogens may be particularly prevalent. Bartonellosis is contracted by handling animals infested with fleas, animal owners and veterinarians are at particular risk [17]. Domestic cats are considered to be the natural reservoir for *Bartonella henselae* and *Bartonella clarridgeiae*, the causative agents of cat scratch disease, and dogs may also be infected with these pathogens [18, 19]. *Ctenocephalides felis felis* is assumed to be the main vector for *B. henselae* and *B. clarridgeiae* [20]. Several studies have reported the presence of *Bartonella* spp. DNA in various flea species suggesting their role as potential vectors [2].

Three species of haemotropic mycoplasmas (also known as haemoplasmas), *Mycoplasma haemofelis*, “*Candidatus* Mycoplasma haemominutum” and “*Candidatus* Mycoplasma turicensis”, have been reported in UK cats [21]. *Mycoplasma haemofelis* is the most pathogenic feline haemoplasma, occasionally causing severe haemolytic anaemia in acute infections [22]. Infections with the other two haemoplasma species may cause a drop in erythrocyte parameters, but these cats do not usually become clinically anaemic unless their health is compromised or they are immunosuppressed [22]. The natural route of transmission of these pathogens between cats is yet to be determined, but the possible role of an arthropod vector is supported by the detection of feline haemoplasma DNA in fleas and ticks collected from cats and/or the environment [23, 24].

The aim of the present study was to undertake a large-scale survey of flea-borne pathogens in fleas collected from cats and dogs in the UK. Increasing levels of pet ownership and increasing urbanisation, along with factors such as climate change and globilisation may, over time, affect the endemicity and prevalance of insect vectors and change the dynamics of pathogen transmission. Therefore, a more detailed understanding of the current prevalence and distribution of flea-borne pathogens is important and should be supported by continued surveillance. This will aid in better education of veterinarians, physicians and public health workers and help develop and implement more effective preventive measures. Such surveillance has been facilitated in recent years by the wide availability of molecular tools, although, given the relatively low prevalances of flea-borne pathogens, large sample numbers are required for statistically meaningful results.

## Methods

### Flea collection and questionnaire

To collect flea samples for the present study, a nationwide campaign was instigated in March 2018 to recruit veterinary practices. Practices that registered an interest in participating were sent a kit, consisting of an inspection protocol, questionnaires, envelopes, sealable bags and flea combs. The protocol instructed veterinary practitioners to select 5 cats and 5 dogs per week at random for four weeks. The randomisation procedure to be adopted was not specified; however, veterinarians were asked to undertake flea inspections for animals where the infestation status was unknown, for example when giving booster injections, routine operations, or when offering free flea checks or at other routine nurse clinics. Each flea check was done using a dampened flea comb. The pet was combed, focusing attention on the parts of the body most likely to harbour fleas: the lower back, tail-head, and posterior and inner thighs. The dampened comb increased the probability of fleas sticking to the comb-teeth long enough to allow capture. At the end of the grooming process, the entire comb was placed in plastic sample bag and sealed. Veterinarians were asked to complete a questionnaire for each animal regardless of whether fleas were found or not, recording the owner address, pet species, breed, sex, neutered status, presence and abundance of fleas, whether the pet had been abroad in the previous two weeks and its insecticidal treatment history. Veterinarians could print and post the questionnaires or submit them online. All flea samples were sent by standard post to the University of Bristol where they were stored at -20 °C.

### Data handling, mapping and flea identification

Questionnaire data were entered into Microsoft Excel and the WGS84 (World Geodetic System) map coordinates of each pet owner’s location was recorded. The geographical program QGIS (Open Source Geospatial Foundation Project. https://qgis.org/en/site/, Version 2.18.2) was used to map the location of samples. Fleas were identified to species level using dichotomous keys [25, 26].

### DNA extraction and amplification

After identification, fleas were transferred into individual micro-tubes and all the fleas of the same species collected from a single pet, were pooled together into what will be described as a ‘sample’. DNA extraction was carried out using a high throughput system, DNeasy 96 Blood & Tissue Kit (Qiagen, Manchester, UK). The flea samples were crushed using micro-pestles in their respective tubes and thoroughly mixed in 180 μl Buffer ATL and 20 μl proteinase K by vortexing. The samples were spun down and incubated overnight at 56 °C to ensure complete tissue lysis. After overnight incubation, the lysates were spun down and all the contents from each tube were transferred to individual wells of a 96 deep-well plate, leaving behind the flea exoskeleton. Further extraction steps were carried out as per the manufacturer’s guidelines. To check the efficacy of the DNA extraction, a conventional PCR targeting the *18S* rRNA gene of fleas [27] was run before running the PCRs for the detection of pathogens.

Flea DNA in extracted samples was detected with conventional PCR that amplified a 1200 base pair (bp) region of the flea *18S* rRNA gene. A master mix was made as follows: 5 μl of 2× GoTaq Hot Start Mastermix (Promega, UK), 0.2 μl of 10 μM each forward (18S-F)/reverse (18S-R) primer mix (Table 1) and 2.8 μl water. Two μl of extracted DNA were then added to 8 μl of master mix in 96 well PCR plates using a high throughput automated pipetting system (epMotion P5073, Eppendorf, Stevenage, UK). Flea DNA and water were used as positive and negative controls, respectively. The thermal cycling protocol consisted of an initial denaturation at 95 °C for 2 min, followed by 40 cycles of 95 °C for 20 s, 56 °C for 20 s and 72 °C for 90 s in a thermal cycler (Biorad T100 thermal cycler, Biorad, Watford, UK). Amplified DNA was subjected to electrophoresis in a 2% agarose gel pre-stained with 0.05 μg/ml ethidium bromide and viewed under ultraviolet light. Positive samples were identified as having a defined band of ~1200 bp on the gel.

**Table 1:** Details of the qPCR/PCR assays used in the study for the detection of pathogen DNA in flea samples

Target species (gene)	PCR primer and probe sequences (5'-3')	Product size (bp)	Reference
Flea (*18S* rRNA)	18S-F: GATCGTACCCACATTACTTG	1200	[27]
	18S-R: AAAGAGCTCTCAATCTGTCA		
*Dipyllidium caninum* (*28S* rRNA)	F: GCATGCAAGTCAAAGGGTCCTACG	653	[48]
	R: CACATTCAACGCCCGACTCCTGTAG		
*Bartonella* spp. (*ssrA*)	F: GCTATGGTAATAAATGGACAATGAAATAA	299	[28]^a^
	R: GGCTTCTGTTGCCAGGTG		
	FAM-ACCCCGCTTAAACCTGCGACG-BHQ1		
*Mycoplasma haemofelis* (*16S* rRNA)	F: GTGCTACAATGGCGAACACA	80	[29]
	R: TCCTATCCGAACTGAGACGAA		
	FAM-TGTGTTGCAAACCAGCGATGGT-BHQ1		
“*Candidatus* Mycoplasma haemominutum” (*16S* rRNA)	F: TGATCTATTGTKAAAGGCACTTGCT	135	[29]
	R: TTAGCCTCYGGTGTTCCTCAA		
	FAM-TTCAATGTGTAGCGGTGGAATGCGT-BHQ1		
“*Candidatus* Mycoplasma turicensis” (*16S* rRNA)	F: AGAGGCGAAGGCGAAAACT	138	[29]
	R: ACGTAAGCTACAACGCCGAAA		
	FAM-CGTAAACGATGGGTATTAGATGTCGGGAT-BHQ1		

^a^The reverse primer has been modified compared to the one described in the paper

### *Bartonella* spp. quantitative PCRs and DNA sequencing

*Bartonella* spp. were detected using a quantitative PCR (qPCR) targeting a fragment of the *ssrA* gene [28] modified as follows: each qPCR consisted of GoTaq Hot Start Mastermix (Promega, Southampton, UK), MgCl_2_ to a final concentration of 4.5 mM, forward and reverse primers (Table 1) at a final concentration of 500 nM each and TaqMan probe (Table 1) at a final concentration of 100 nM, 5 μl of template DNA and water to a final volume of 25 μl. The thermal cycling protocol consisted of an initial denaturation at 95 °C for 2 min and 40 cycles of 95 °C for 15 s and 60 °C for 30 s (Agilent MX3005P qPCR, Agilent, Stockport, UK). Fluorescence data were collected at 520 nm at the end of each annealing/extension step. A positive control sample (of known copy number) and negative control (water) were included on each plate. All samples positive for *Bartonella* spp. were prepared using a Nucleospin® 96 PCR Clean-up Core Kit (Macherey-Nagel, Düren, Germany) and submitted to a commercial sequencing laboratory (DNA Sequencing & Services, MRC I PPU, School of Life Sciences, University of Dundee, UK) for DNA sequencing using Applied Biosystems Big-Dye Ver 3.1 chemistry on an Applied Biosystems model 3730 automated capillary DNA sequencer.

### *Dipyllidium caninum* PCR

Conventional PCR was used to amplify a 653 bp region of the *28S* rRNA gene of *D. caninum* in the flea samples. A master mix was made as follows: 5 μl of 2× GoTaq Hot Start Mastermix (Promega, Southampton, UK), 0.2 μl of 10 μM each forward and reverse primer (Table 1) and 2.8 μl water. Two μl of extracted DNA were then added to 8 μl of master mix in 96-well PCR plates using a high throughput automated pipetting system (epMotion P5073, Eppendorf, Stevenage, UK). *Dipylidium caninum-*positive DNA samples (Clinvet, South Africa, www.clinvet.com) were used as positive controls and water as a negative control. The thermal cycling protocol consisted of an initial denaturation at 95 °C for 2 min, followed by 40 cycles of 95 °C for 20 s, 66 °C for 5 s and 72 °C for 30 s in a thermal cycler (Biorad T100 thermal cycler). Agarose gel electrophoresis was used to visualise target amplicons. Positive samples were identified from a defined band of ~650 bp on the gel.

### Haemoplasma species qPCRs

Feline haemoplasma DNA was detected using individual species-specific qPCRs targeting the *16S* rRNA gene of *M. haemofelis*, “*Ca.* M. haemominutum” and “*Ca.* M. turicensis” (Table 1), as previously described [29]. The canine haemoplasma *M. haemocanis* is also detected by the *M. haemofelis* qPCR. The qPCR assay for each species consisted of GoTaq Hot Start Mastermix (Promega), MgCl_2_ to a final concentration of 4.5 mM, forward and reverse primers (for each species as shown in Table 1) at a final concentration of 200 nM each, TaqMan probe (for each species as shown in Table 1) at 50 nM, 2 μl of template DNA and water to a final volume of 10 μl. A positive control, *M. haemofelis* (of known copy number) and negative control (water) were included on each plate. The thermal cycling conditions were identical to those described above for the *Bartonella* spp. qPCR.

## Results

### Species abundance and distributions

**Fig. 1:**
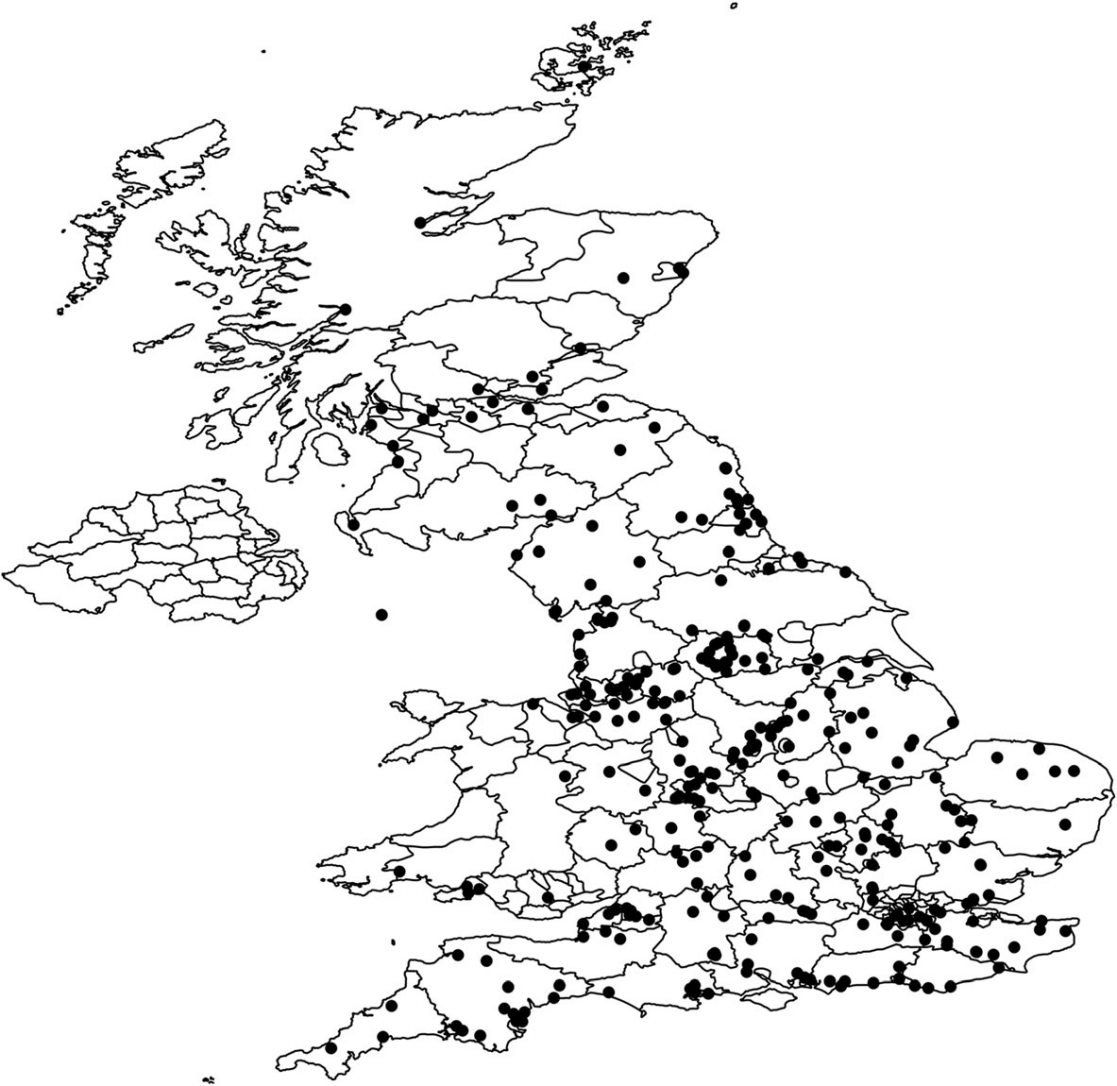
Distribution of 326 veterinary practices that participated in the survey. Black dots indicate the postcode location of the veterinary practice

**Fig. 2:**
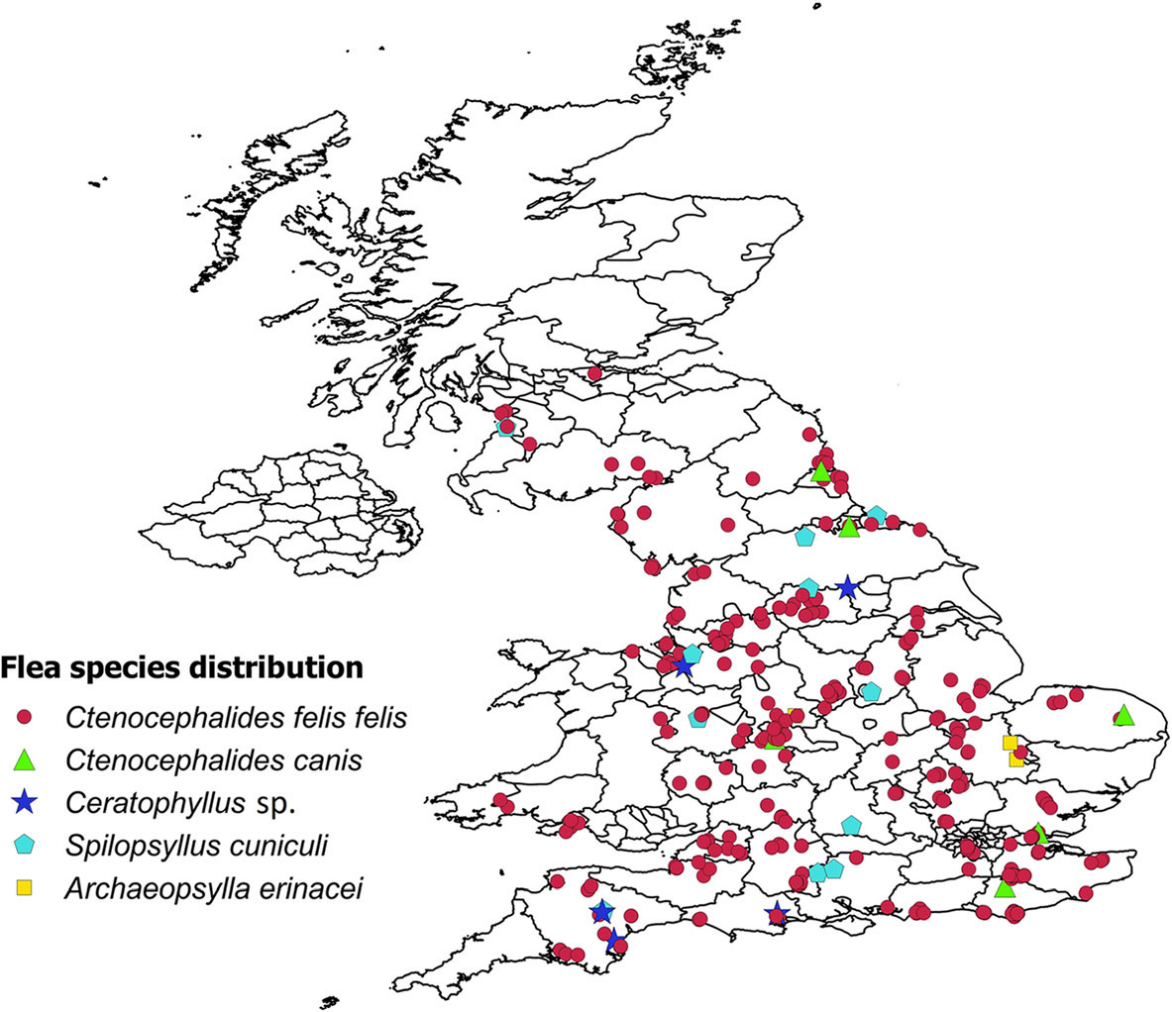
Distribution of different flea species collected from dogs and cats in the UK. The different symbols indicate the postcode location of the pet owner or the veterinary practice

A total of 326 veterinary practices from across the UK participated between April and June 2018 (Fig. 1); during this time a total of 1627 animals (cases) were examined. For 1475, of these a questionnaire was submitted that was wholly or at least partially completed. Among the 1475 animals with questionnaire returns, 812 were cats and 662 were dogs and in one instance no information about the host species was present on the otherwise completed questionnaire (Table 2). Three hundred and twenty-three of the 1475 cases (21.9% ± 95% CI 2.1%) with wholly or partially completed questionnaire records were infested with fleas. Of the 812 cats examined, 228 (28.1% ± 95% CI 3.09%) were infested with fleas and of the 662 dogs examined, 95 (14.4% ± 95% CI 2.67%) carried fleas (Table 2). Amongst the 228 flea infested cats, 92% carried the cat flea *C. f. felis*, 1.3% the dog flea *C. canis*, and 4% the rabbit flea *S. cuniculi*. Among the 95 flea-infested dogs, 90% were infested by cat fleas, 3% by dog fleas, and 2% by rabbit fleas. Small numbers of both cats and dogs were infested with hedgehog, *A. erinacei*, or hen, *Ceratophyllus* spp. fleas (Table 2). The median number of fleas collected per animal was 1, although a maximum number of 89 fleas were reported from a cat and 18 from a dog. Flea samples from two cases were too damaged to be identified. The flea species from different cases were widely distributed although, notably, relatively few cases were reported from northern areas, particularly from Scotland (Fig. 2). An additional 152 flea samples were sent without any questionnaire records at all, so no information about the host species from which they were collected was available (Table 2). These fleas were included in the pathogen analysis but could not be included in the calculation of prevalence by host. The data for these 152 flea samples is reported separately from the other samples.

**Table 2:** The number of cats and dogs examined during the survey, the number with or without fleas and number infested with various species of fleas collected from each pet species. In some cases flea identification was not possible (No ID) because the specimens were too damaged, while in others the host species was not recorded (no records) by the veterinary practice

Pet species	No. of examined animals with records	Fleas absent *n* (%)	Fleas present *n* (%)	*C. felis felis*	*C. canis*	*S. cuniculi*	*A. erinacei*	*Ceratophyllus* spp.	No ID
Cat	812	584 (71.9)	228 (28.1)	210 (25.6)	3 (0.4)	9 (1.1)	2 (0.25)	3 (0.4)	1
Dog	662	567 (85.6)	95 (14.4)	86 (13.0)	3 (0.5)	2 (0.3)	1 (0.2)	2 (0.3)	1
No records	–	–	152	133	3	8	3	4	0
Total	1474	–	475	429	9	19	6	9	2

### Prevalence and geographical location flea-borne pathogens

DNA samples from 470 pooled flea samples were analysed for *D. caninum*, *Bartonella* spp. and haemoplasma species. Of the 470 pooled flea samples, 66 were positive for the DNA of at least one of these pathogens (14% ± 95% CI 3.14%).

Fifty-three of the 470 pooled flea samples (11.3% ± 95% CI 2.85%) were found to be positive for *Bartonella* spp. DNA. Among these, 35 were collected from cats and 4 from dogs, 14 flea samples did not have any record of their host species. Forty-nine of the *Bartonella* spp. positive samples were from *C. f. felis*, 3 were from *S. cuniculi* and one was from an *A. erinacei*. Thirty-two of the infected *C. f. felis* samples were collected from cats, whereas as only 4 samples were collected from dogs; two *Bartonella*-positive samples from *S. cuniculi* and one from *A. erinacei* were also collected from cats. Among the 14 flea samples without any host record, 13 were from *C. f. felis* and one was from *S. cuniculi*.

**Fig. 3:**
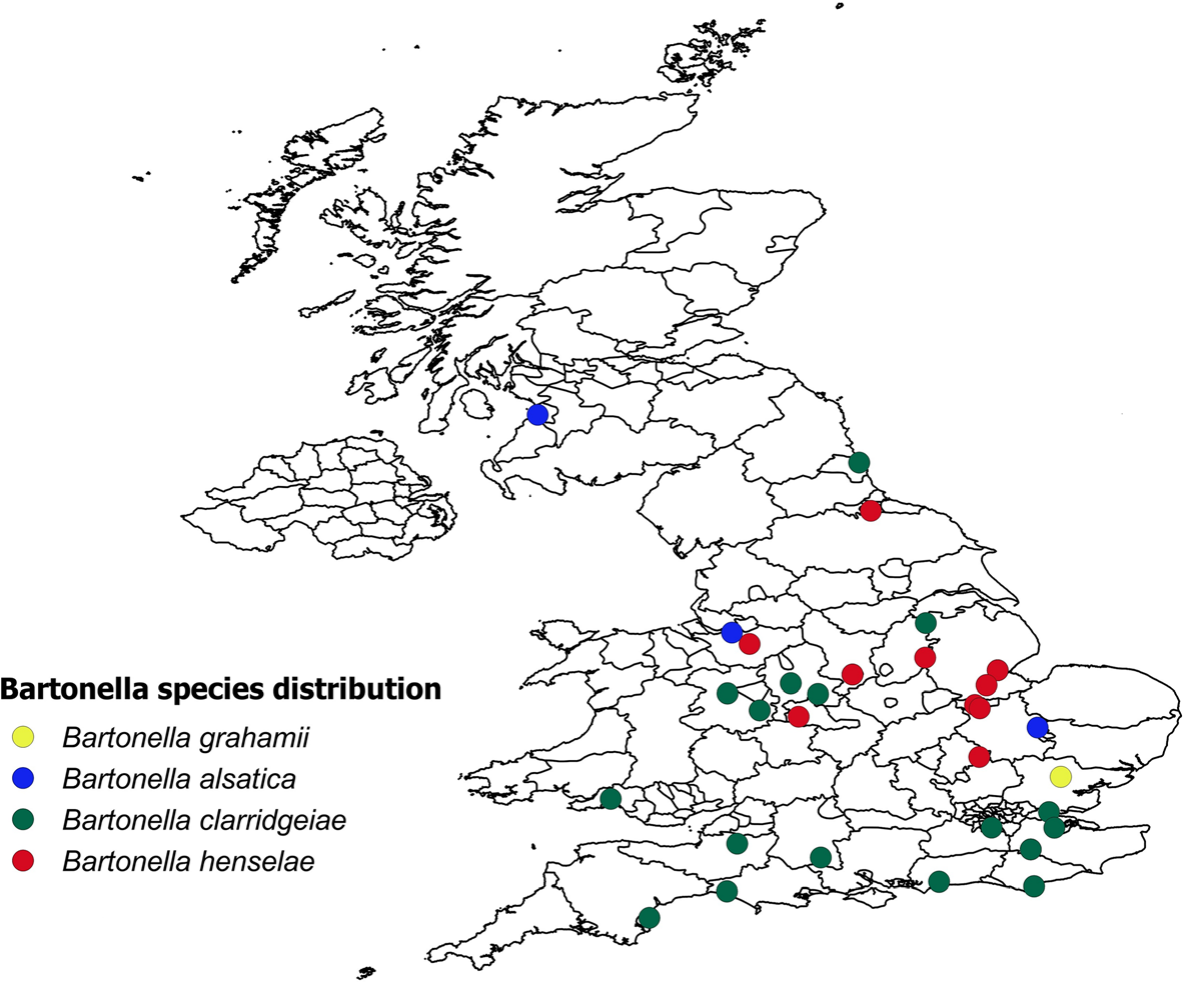
Distribution of *Bartonella* species detected in fleas collected from dogs and cats in the UK. The circles indicate the postcode location of the pet owner or the veterinary practice

Among the 53 *Bartonella* spp. qPCR positive samples, 4 could not be sequenced and the remaining 49 samples belonged to four different *Bartonella* species. Thirty-two were from cats and 3 were from dogs and 14 had no record of the host species from which the fleas were collected (Table 3). Seventeen samples were found to be *B. henselae*, 27 were *B. clarridgeiae*, and these belonged to two different strains (Table 3). Four samples were *B. alsatica*, and 1 was *B. grahamii*. Fifty of the 53 fleas carrying *Bartonella* spp. were *C. f. felis*, two were *S. cuniculi* and 1 was *A. erinacei*. The *S. cuniculi* and *A. erinacei* flea samples were positive for *B. alsatica*. Most of the samples positive for *Bartonella* spp. came from central and southern England (Fig. 3).

**Table 3:** Number of different *Bartonella* species detected in flea samples of different species in different cat or dog hosts. In some cases, the host species was not recorded (no record) by the veterinary practice

*Bartonella* spp.	No. of infected flea samples	qPCR Ct values (range)	GenBank ID	Sequence homology (%)	Flea species	Host species
*B. grahamii* strain as4aup	1	36.94	HG519007.1	99	*C. f. felis*	Cat
*B. alsatica* strain IBS 382	4	22.7–33.17	JN029776.1	95–99	*S. cuniculi* (*n* = 2); *A. erinacei* (*n* = 1); *C. f. felis* (*n* = 1)	Cat (*n* = 3); no record (*n* = 1)
*B. clarridgeiae* strain 73	24	18.1–36.8	HG519012.1	97–99	*C. f. felis*	Cat (*n* = 15); dog (*n* = 3); no record (*n* = 6)
*B. clarridgeiae*strain Rc_AL817-1	3	35.9–37.5	KY417894.1	96–99	*C. f. felis*	Cat (*n* = 2); no record (*n* = 1)
*B. henselae* Houston-I	17	21.0–36.3	CP020742.1	94–100	*C. f. felis*	Cat (*n* = 11); no record (*n* = 6)

Fourteen of the 470 (3% ± 95% CI 1.53%) pooled flea DNA samples were found to be positive for *D. caninum* DNA. A positive result was indicated by a single band of ~650 bp by gel electrophoresis and all positive samples had bands of greater than 25 ng per 5 μl loaded. The distribution of the positive samples was widespread, although they were mostly concentrated in southern England and none of the fleas in northern England or Scotland were found to be positive for the DNA of this cestode. Among the 14 positive flea samples, 13 were *C.f. felis* and one was *C. canis*. Ten of the infected *C. f. felis* samples were collected from cats and one from a dog, the other 3 positive flea samples did not have a record of the host species.

The species-specific qPCRs for haemoplasma species found that only 3 flea samples were positive for *M. haemofelis* or *M. haemocanis*, all of which were found in *C. f. felis* fleas, 2 of which were collected from cats and one had no record of the host species. Thus, it would be expected that the infecting species in these flea samples was *M. haemofelis.* None of the flea samples were found to be positive for either “*Ca.* M. haemominutum” or “*Ca.* M. turicensis”.

Three flea samples were positive for both *D. caninum* and *Bartonella* spp. One flea sample was positive for both *Bartonella* spp. and *M. haemofelis* or *M. haemocanis*.

## Discussion

In the present study, the flea infestation rate was high for both cats (28.1 ± 3.09%) and dogs (14.4 ± 2.67%), but both host species were almost equally likely to carry the cat flea, *C. f. felis*. Relatively few dog fleas were found, but these did occur more frequently on dogs. Relatively low numbers of rabbit, hedgehog and hen fleas were found, with no *Pulex irritans* identified. The protocol for recruitment to the study highlighted the need for veterinarians to seach pets brought into the practice for reasons other than flea infestation, suggesting that if known flea-infested animals were included, the prevalence would be even higher. Previous studies have shown that the flea species primarily associated with cats is *C. f. felis*; however, for dogs the results are more variable. *Ctenocephalides felis felis* was shown to be the most common species of flea on domestic dogs in the UK [30–32]. High levels of infestation of dogs by *C. f. felis* have also been reported in North America [33], Denmark [34] and Finland [35]. In contrast, *C. canis* was found to be more common on dogs in the UK than *C. f. felis* [36], as was the case in Ireland [37, 38]. In a further study in Ireland of 193 cases, 90% of all infestations on cats were with *C. f. felis*; only a single cat was found to be infested by *C. canis*. In contrast, in the dogs 17.5% were infested by *C. f. felis* and 75.7% by *C. canis* [39]. A preponderance of *C. canis* on dogs has also been reported in New Zealand [12], Denmark [40] and the Republic of Korea [41]. In a study of fleas infesting kennel dogs from two localities in Israel, a total of 355 fleas were collected from 107 dogs, of which 74.8% had *C. canis*, 63.6% had *C.f. felis*, 14.0% had *P. irritans* and one animal had *Xenopsylla cheopis* [42].

Flea borne pathogens in cats and dogs can result in significant levels of clinical disease and are of concern for veterinarians and pet owners [16]. In this study, 11% ± 2.85% of the pooled flea samples of different species and collected from both cats and dogs were found positive for *Bartonella* spp. Four different *Bartonella* species were found in the positive flea samples, and these samples were distributed mostly around central and southern UK. This could possibly be because fewer veterinary practices were recruited in northern parts of the UK, but equally may reflect a more southerly distribution of fleas since relatively few cases were submitted from northern England or Scotland; further spatial analysis is required to quantify this trend. The effects of infection by *Bartonella* spp. may range from asymptomatic to fatal. The most common zoonotic species is *B. henselae*, for which cats are the major natural reservoir. Fleas, *C. f. felis* in particular, are the known vectors for *B. henselae*, *B. clarridgeiae* and *Bartonella koehlerae* [17]. The results of this study highlight the anticipated strong association between *Bartonella* spp. and *C. f. felis* feeding on cats. Evidence of exposure to *Bartonella* spp. in cats has been found in many countries, particularly in regions with high humidity [43, 44]. In cats, bartonellosis can result in lymphadenopathy, endocarditis, myocarditis and hyperglobulinemia. However, most cats infected with a *Bartonella* spp. will show no clinical signs [45]. Since *B. henselae* survives at least nine days in flea faeces, flea control is imperative to attempt to reduce the risk of infection of other cats, dogs, or people [9, 46]. In this study *Bartonella* spp. were also found in fleas collected from dogs, which has also been noted in other studies [42] where 7.8% of pooled flea samples from kennel dogs from two localities in Israel were positive for *Bartonella* DNA. It should be noted that qPCR for *Bartonella* spp. detection alone may not be the most sensitive approach, which may be achieved more accurately using a combination of conventional and nested PCRs from blood and liquid culture samples [1]. Hence, the prevalence figures reported here may be an underestimate.

*Dipylidium caninum* is a common intestinal cestode parasite of dogs and cats. The onchospheres are contained in egg packets, each with about 20 eggs, and these are either expelled by the active segment or released by its disintegration. After ingestion by a larval flea intermediate host, the onchospheres travel to its haemocoel, where they develop into cysticercoids. The final host is infected by ingestion of the flea containing the cysticercoids. Occasionally humans have become infected following ingestion of the saliva of infected pets [12]. The potential zoonotic transmission and wide geographical range emphasize the importance of protecting dogs and cats from *D. caninum*. Routine treatment may be an effective approach to tapeworm management; however, the brief pre-patent period and lack of residual activity of most treatments means that reinfection may occur rapidly. Hence effective and persistent flea control is an important element of any tapeworm management regime. A relatively low prevalence of *Dipylidium* has been reported in studies using coproscopy. For example, in a study of 2775 dog faecal samples from the Lazio Region of central Italy (1156 from households and 1619 from shelter dogs) only 0.1% were found to be infected [16]. In Greece, in a study of the faeces from 1150 cats, a prevalence of 0.2% was detected [47]. However, the poor sensitivity of coproscopy means that faecal analysis is likely to significantly underestimate infection by *Dipylidium*. In PCR analyses of flea samples from 435 cats, 4.37% of fleas were found to be infected with *D. caninum* and in fleas from 396 dogs, 9.1% carried *Dipylidium* [48]. In the present study, the prevalence of *D. caninum* was similar, with 14 of the 470 (3 ± 1.53%) flea DNA samples found to be positive for *D. caninum* DNA but the majority of the *D. caninum-*positive flea samples were collected from cats and only one was collected from a dog. Of particular interest is the recent finding of two distinct genotypes of *D. caninum* in dogs and cats, suggesting that two distinct species may be present [49], but this was not investigated in the present study.

Like *Bartonella* spp., the DNA of haemoplasmas has been amplified from the blood of cats in many regions of the world [44], with *M. haemofelis* usually considered to be the most pathogenic species [22, 50]. The present study found only 3 flea samples positive for *M. haemofelis* or *M. haemocanis* DNA, and it was not possible to differentiate between these two haemoplasma species as their *16S* rDNA sequences are near identical [51]. A similarly low prevalence of *M. haemofelis* DNA was found in ticks collected from pets in the UK [24] and no haemoplasma DNA was found in fleas collected from cats in southern Italy [44]. In contrast in a study involving 1585 cats, found 2.8% cats to be positive for *M. haemofelis* and 1.7% positive for “*Ca.* M. turicensis” [29]. The zoonotic importance of feline haemoplasmas is still being questioned [52]. Clinical signs of disease depend on the degree of anaemia, the stage of infection and the immune status of infected cats. Direct transmission may occur with the hemoplasmas, and studies have found some of the agents in saliva [53] and that subcutaneous inoculation of hemoplasma-containing blood resulted in infection transmission [54]. Infection does not necessarily result in clinical disease and in some cases healthy cats can also be positive for haemoplasma DNA in blood [29, 55, 56] and so PCR assay results do not always correlate well with clinical illness.

The methods used to evaluate the prevalence of flea-borne pathogens and the role of fleas as vectors vary in their sensitivity and each is subject to different biases. The use of host blood samples can be problematic. In a study of ectoparasites on cats and vector-borne pathogens in feline blood samples in southern Italy which used qPCR there was little agreement between serological and molecular results in individual cats and the presence of ectoparasites, with the exception of *B. clarridgeiae* and *B. henselae* [44]. The authors argued that the ability to detect pathogens in the blood depends strongly on the immunological sensitivity of the host; in addition, the bacteraemia of some pathogens is transient, lasting only a few hours. This makes it difficult to detect pathogens in the blood and requires samples to be taken at very specific time points and the use of highly sensitive molecular tools; the use of serology may therefore underestimate the prevalence of pathogens. The present study investigated the presence of pathogen DNA amplified from fleas but, even though fleas tend to remain on the same host once acquired, the blood in the gut could potentially have come from more than one host, particularly where pets live in close contact in the same household; the presence of pathogen DNA in the gut alone also does not demonstrate vectoral competence [24]. There is a possibility of carryover of the pathogen DNA from the host blood, especially where the Ct values of the qPCR is higher than 36 cycles. The detection of *D. caninum* DNA from adult fleas collected off a host also requires careful interpretation because, although it is highly indicative, it represents the potential for infection rather than infection itself, since the adult flea would need to be ingested by the grooming host to result in infection. As a result, ideally, a combination of epidemiological indicators is required to establish the true prevalence and the role of arthropod vectors in the transmission of pathogens [57].

## Conclusions

The present study indicates that veterinary practices were able to find fleas in a quarter of cats and one sixth of the dogs examined during the study period and the flea samples were found to be positive for a range of infectious agents; in particular the study highlights the relatively high prevalence of *Bartonella* spp., particularly in central and southern areas, which is of concern for both animal welfare and human health. The study highlights the ongoing need to educate pet owners about the effects of both flea infestation but also the pathogen risks these fleas present.

## Abbreviations

95% CI: 95% confidence interval
qPCR: Quantitative polymerase chain reaction
Ct: Cycle threshold
ng: Nano gram
μl: Micro litre
bp: Base pair
nM: Nano molar
°C: Degree centigrade
